# ^1^H, ^15^N, ^13^C resonance assignment of the human CD44 cytoplasmic tail (669–742)

**DOI:** 10.1007/s12104-018-9861-0

**Published:** 2018-11-24

**Authors:** Benjamin Frühbauer, Borja Mateos, Robert Konrat

**Affiliations:** Max F. Perutz Laboratories, Department of Computational and Structural Biology, University of Vienna, Campus Vienna Biocenter 5, 1030 Vienna, Austria

**Keywords:** CD44, Intrinsically disordered region, Cell–matrix adhesion, Cell migration, Extracellular matrix

## Abstract

CD44 is a universally and abundantly expressed single-pass type I protein that spans the cytoplasmic membrane and is considered the principal receptor for hyaluronan in the extracellular matrix. CD44 exerts a multitude of biological functions, especially in cell adhesion and migration, and its deregulation has several pathological implications, including a putative role in cancer cell dissemination. Here we report the NMR chemical shift assignment of the recombinant intrinsically disordered CD44 cytoplasmic region (669–742).

## Biological context

CD44 is the principal transmembrane receptor of the glycosaminoglycan hyaluronan (HA), which can be found in abundance in the extracellular matrix (Toole 2004). It exists in several splice variants and is heavily modified after its translation, especially by glycosylation of the extracellular stem part (Zöller 2011). The functionality of CD44 seems manifold, though involved to a large extent in several aspects of the immune system, such as lymphocyte activation (Degrendele et al. 1997), rolling interaction and homing (Gál et al. 2003; Suzuki et al. 2015), as well as in hematopoiesis and adhesion via cell–cell and cell–matrix interactions in general (Perschl et al. 1995; Ponta et al. 2003; Wolny et al. 2010). CD44, however, has attracted particular interest due to its prominent role in several pathologies, especially in the context of cancer initiation and metastasis (Naor et al. 2009; Zöller 2011; Goldman et al. 2015; Wang et al. 2018).

It was discovered early on, that the functionality of the receptor is influenced by post translational modifications that target the intrinsically disordered C-terminal region of the protein, that is found on the cytoplasmic side of the cell. It had been shown, that specific phosphorylation events can temper with migration and adhesion of CD44 expressing cells on HA-containing surfaces (Puré et al. 1995; Peck and Isacke 1998; Gál et al. 2003), which has been suggested to be attributable to phosphorylation-dependent interactions between the CD44 cytoplasmic region and the ezrin–radixin–moesin protein family that connect the actin cytoskeleton with this membrane receptor (Yonemura et al. 1998; Mori et al. 2008; Jokela et al. 2015). Moreover, it has been previously shown that both the extracellular region and the cytoplasmic tail are proteolytically processed by ADAM10 and γ-secretase, respectively (Lammich et al. 2002; Hartmann et al. 2015). In the present letter, we present the NMR assignment of the CD44 cytoplasmic tail generated after γ-secretase cleavage of full-length CD44.

## Methods and results

### Sample preparation

**Fig. 1:**
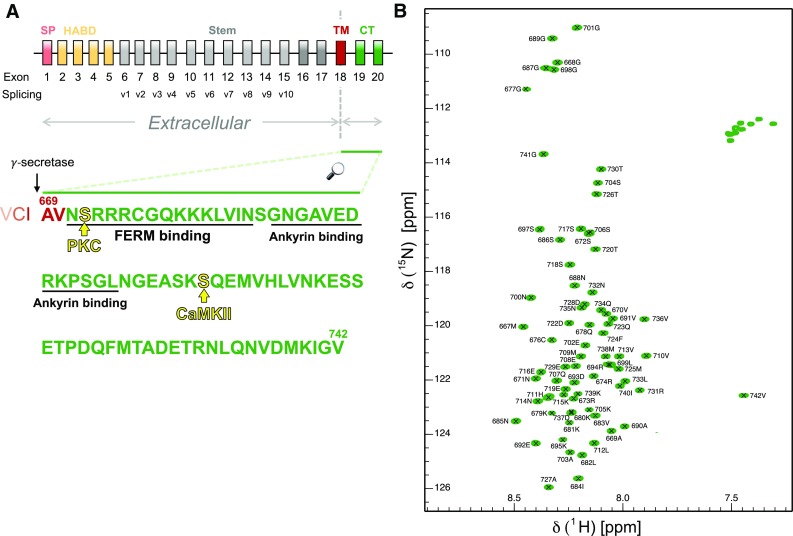
**a** Schematic organization of genomic CD44 exons. The cytoplasmic tail resulted after γ-secretase processing comprises residues 669–742 (Uniprot: https://www.uniprot.org/uniprot/P16070). Protein primary sequence with the main binding regions (underlined) and phosphorylation sites (yellow) indicated. Additional four residues (GAMG) are in the final sample due to construct design in pETM-11 for TEV cleavage. **b**^1^H–^15^N HSQC spectrum of CD44_669–742_ at pH 6.5 and 293 K

The pETM-11 expression vector (Dümmler et al. 2005) for human CD44 cytoplasmic codon-optimized cDNA was acquired from GenScript®, coding for a 78-residue construct comprising four non-canonical residues, followed by the two final residues of CD44’s transmembrane domain and the 72 canonical residues of the cytoplasmic domain, fused to an N-terminal His_6_-tag with a TEV-cleavage site to allow separation of the expressed construct from the purification tag, as shown in Fig. 1. Expression of ^15^N/^13^C labeled protein was conducted in the *Escherichia coli* BL21 (DE3) strain (New England Biolabs) in minimal medium for isotopic labeling according to Bracken et al. (2001). Bacterial cultures were expanded in a fourfold excess of LB (4 L per liter of expression culture) until an OD_600_ of 0.8 was reached, where after the cells were harvested by centrifugation for 15 min at 4000 rpm and 4 °C and then resuspended in minimal medium. Expression was induced by addition of IPTG to a final concentration of 1 mM after the culture recovered for 10 min at 37 °C and appropriate agitation. After induction the culture was incubated for expression at 28 °C and appropriate agitation for 18 h, followed by harvesting of the cells by centrifugation at 4000 rpm and 4 °C for 20 min. The bacterial pellet was resuspended in a 50 mM Tris–HCl washing buffer (300 mM NaCl, pH 8), 40 mL per liter of expression culture, supplemented with Halt™ Protease Inhibitor Cocktail (Thermo Scientific™) and 2 mM β-mercaptoethanol. Cells were lysed via sonication, followed by centrifugation at 18,000 rpm and 4 °C for 40 min after which the supernatant was heated to 80 °C for 10 min to facilitate precipitation of heat-sensitive impurities which were separated from the sample by subsequent centrifugation at 18,000 rpm and 4 °C for 40 min. The resulting supernatant was loaded onto a 5 mL HisTrap™ High Performance (GE Healthcare) affinity chromatography column pre-loaded with Ni^2+^ and equilibrated with 50 mM Tris–HCl washing buffer. The column was then washed with 3 column volumes of 50 mM Tris–HCl low imidazole buffer (1000 mM NaCl, 20 mM imidazole, pH 8) and re-equilibrated in 50 mM Tris–HCl washing buffer prior to elution by 50 mM Tris–HCl elution buffer (300 mM NaCl, 500 mM imidazole, pH 8) using a successively increasing gradient. All collected fractions containing the CD44 cytoplasmic region were pooled and the buffer was exchanged to a × 1 PBS TEV cleavage buffer (140 mM NaCl, 2.7 mM KCl, 10 mM Na_2_HPO4, 1.8 mM KH_2_PO_4_, pH 7.5) supplemented with 1 mM EDTA and 1 mM DTT using a 3 kDa cut-off Amicon® Ultra Centrifugal Filter (Merck). 0.5 mg TEV protease were added for each liter of original expression culture and incubated for 30 min at room temperature, followed by slow mixing at 4 °C for 16 h. The cleaved construct was loaded on a HiLoad® 16/600 Superdex® 75 pg column pre-equilibrated with 50 mM sodium phosphate buffer (50 mM NaCl, 0.02% NaN_3_, pH 6.5) and size exclusion chromatography was performed using the same buffer. The collected fractions of the CD44 cytoplasmic region were pooled and concentrated using a 3 kDa cut-off Amicon® Ultra Centrifugal Filter (Sigma) until a reasonable concentration of roughly 0.7–0.9 mM was reached, estimated via BCA Protein Assay (Thermo Scientific™) and measurement of free thiol via 5,5′-dithio-bis-[2-nitrobenzoic acid] reaction (Thermo Scientific™). NMR samples were supplemented with 1 mM DTT before measurement.

### NMR experiments

**Fig. 2:**
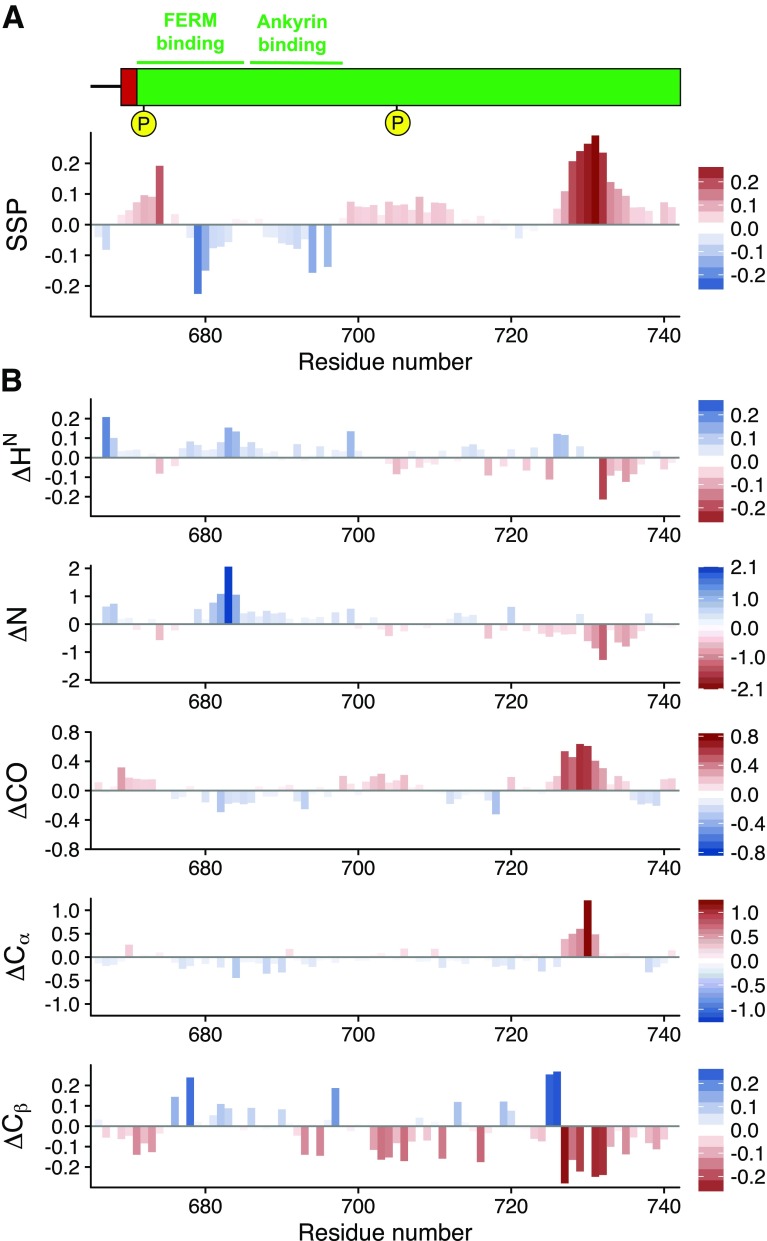
**a** SSP scores (Marsh et al. 2006) for CD44 cytoplasmic region using Cα and Cβ chemical shifts. A positive value represents a tendency to form an α-helix and negative values indicate extended or β-sheet propensities. **b** Secondary shifts extracted from comparing experimental shifts with random-coil values calculated with POTENCI (Nielsen and Mulder 2018). Red bars indicate propensity for α-helix and blue bars for β-sheet. Phosphorylation sites are indicated in yellow

NMR data was acquired at 293K on a Bruker NEO 600 MHz spectrometer equipped with a TXI-probehead. The backbone assignments were obtained using BEST-type versions of HNCACB, HN(CO)CACB, HNCO, and HN(CA)CO experiments (Schanda et al. 2006; Lescop et al. 2007). Data acquisition followed a non-uniform sampling strategy with 25% (total 64^*^ × 128^*^ = 8192 hypercomplex points) equivalent to 2048 FID for all three-dimensional experiments. Reconstruction was carried out using multi-dimensional decomposition method with mmdNMR (Kazimierczuk and Orekhov 2011; Orekhov and Jaravine 2011) within Topspin 4.0.1. Assignment of the CD44 cytoplasmic region resonances was conducted using the CCPNmr software package (Vranken et al. 2005). The secondary structure propensity (SSP) score was estimated as proposed by Marsh et al. (2006) (Fig. 2a) and using random-coil shifts extracted from POTENCI (Nielsen and Mulder 2018) (Fig. 2b).

### Extent of assignment and data deposition

Shown in Fig. 1 is the ^1^H–^15^N HSQC spectrum, exhibiting a relatively narrow proton chemical shift dispersion corresponding well with the expected values for an intrinsically disordered protein. Additional evidence for this is provided in Fig. 2b, showing only relatively small deviations from random coil for most parts of the protein. The same analysis, however, indicates the presence of a slight helical propensity at the C-terminus, which may be an interesting part for future analyses due to putative interaction sites that have been proposed to be localized within this stretch of the region (Jokela et al. 2015), which might allow observations of secondary structure formation upon binding.

Signal dispersion was well provided in most cases, allowing for a rather clear assignment of the observed resonances. Almost all putative residues could be assigned, except for two residues at the N-terminus, Arg675 and Lys681. In total 95.7% of backbone atoms were assigned, which, in more detail corresponds to 93.5% of ^15^N, 97.3% of ^1^HN, 96.1% of ^13^C′, 97.4% of ^13^Cα and 97.1% of ^13^Cβ resonances. The ^1^H, ^15^N and ^13^C chemical shifts have been deposited in the BioMagResBank (http://www.bmrb.wisc.edu/) under the BMRB accession number 27625.
